# GaAs Nanomembranes in the High Electron Mobility Transistor Technology

**DOI:** 10.3390/ma14133461

**Published:** 2021-06-22

**Authors:** Dagmar Gregušová, Edmund Dobročka, Peter Eliáš, Roman Stoklas, Michal Blaho, Ondrej Pohorelec, Štefan Haščík, Michal Kučera, Róbert Kúdela

**Affiliations:** Institute of Electrical Engineering, Slovak Academy of Sciences, Dúbravská Cesta 9, 841 04 Bratislava, Slovakia; edmund.dobrocka@savba.sk (E.D.); peter.elias@savba.sk (P.E.); roman.stoklas@savba.sk (R.S.); michal.blaho@savba.sk (M.B.); ondrej.pohorelec@savba.sk (O.P.); stefan.hascik@savba.sk (Š.H.); michal.kucera@savba.sk (M.K.); robert.kudela@savba.sk (R.K.)

**Keywords:** nanomembrane, hybrid integration, GaAs, InGaAs channel, epitaxial lift-off, HEMT, van der Waals

## Abstract

A 100 nm MOCVD-grown HEMT AlGaAs/InGaAs/GaAs heterostructure nanomembrane was released from the growth GaAs substrate by ELO using a 300 nm AlAs layer and transferred to sapphire. The heterostructure contained a strained 10 nm 2DEG In_0.23_Ga_0.77_As channel with a sheet electron concentration of 3.4 × 10^12^ cm^−2^ and Hall mobility of 4590 cm^2^V^−1^s^−1^, which was grown close to the center of the heterostructure to suppress a significant bowing of the nanomembrane both during and after separation from the growth substrate. The as-grown heterostructure and transferred nanomembranes were characterized by HRXRD, PL, SEM, and transport measurements using HEMTs. The InGaAs and AlAs layers were laterally strained: ~−1.5% and ~−0.15%. The HRXRD analysis showed the as-grown heterostructure had very good quality and smooth interfaces, and the nanomembrane had its crystalline structure and quality preserved. The PL measurement showed the nanomembrane peak was shifted by 19 meV towards higher energies with respect to that of the as-grown heterostructure. The HEMTs on the nanomembrane exhibited no degradation of the output characteristics, and the input two-terminal measurement confirmed a slightly decreased leakage current.

## 1. Introduction

Almost three decades have passed since Boeck and Borghs published their paper on heteroepitaxy versus epitaxial lift-off techniques [1], inspired by the seminal papers of Yablonovitch on epitaxial lift-off (ELO) [2,3]. Since then, a large number of scientific publications have been released regarding thin film epitaxy and film transfer to a variety of host substrates [4]. These publications show that ELO and related techniques are an outgrowth of mature epitaxial growth techniques, such as metal-organic chemical vapor deposition (MOCVD) and molecular beam epitaxy (MBE); they inherently need each other for future microelectronics and nanoelectronics progress.

Although many various two-dimensional materials, such as graphene, MoS_2_, WS_2_, and WSe_2_ [5,6], are intensively studied at present, long well-established thin III-V heterostructures with high-mobility two-dimensional electron gases (2DEG) are exceptionally appropriate for ELO [2,7,8] and for emerging break-through three-dimensional hybrid microelectronic and nanoelectronic technologies [9,10].

In the early years, ELO was mainly used for the separation of relatively thick layers (up to 800 nm and more), and supporting organic layers were very often used in the separation process to increase the sacrificial AlAs etching efficiency and to facilitate the transfer of separated layers [11].

Currently, by contrast, very (ultra) thin, high-quality, single crystalline inorganic semiconductor nanomembranes, with thickness that matches the length scales of important quantum physical processes, are released from their growth substrates and transferred to host substrates. This allows for studies of the basic physics of such nanomembranes [12]. The nanomembranes are released using highly selective etching processes, which utilize sacrificial layers of semiconductor materials, mostly (but not only) AlAs [12,13]. The nanomembranes are attached by van der Waals forces to foreign substrates, such as sapphire, GaN, and plastic flexible substrates [14], and they are used to prepare hybrid equivalents of devices that cannot be produced monolithically [15].

We pursue the hybrid integration of MOCVD-grown III-V- and III-N-based devices, in which GaAs-based heterostructure transistors with excellent dc and high-frequency properties are integrated with devices based on nitride semiconductors on host substrates.

The motivation for our work was to prepare flexible devices based on high electron mobility 2DEG III-V structures. Very thin nanomembranes are extremely flexible, but they can exhibit a low mobility because electrons are increasingly scattered due to interactions at both nanomembrane surfaces. Moreover, the technology of such devices is much more difficult than that of thicker ones. However, 2DEG III-V heterostructures as thin as 100 nm allow for the processing of nanomembranes with mobilities that are comparable with those of the monolithic heterostructures. As such, nanomembranes are very flexible; they can stick to various substrates with van der Waals forces.

## 2. Materials and Methods

This paper reports on the growth, ELO, and transfer of an AlGaAs/InGaAs/GaAs high electron mobility transistor (HEMT) heterostructure to sapphire. The as-grown heterostructure and transferred nanomembrane heterostructure were characterized by high-resolution X-ray diffraction (HRXRD), photoluminescence (PL), and scanning electron microscopy (SEM), and they were used to produce HEMTs, whose performance was studied. The heterostructure contained a strained 10 nm 2DEG In_0.23_Ga_0.77_As channel, which makes the ELO process difficult. This work is inspired by previous studies on hybrid transistor technologies, such as [16,17,18,19,20].

The 2DEG AlGaAs/InGaAs/GaAs heterostructure used in this experiment was designed with the intention to test the limits of heterostructure membrane lift-off and transfer. Therefore, the heterostructure was very thin. Its thickness after separation was nominally ≈ 100 nm. In addition, it was appropriate for the preparation of good-quality HEMTs, which are used to evaluate the quality of nanomembrane transfer by looking into the device transport properties.

The 2DEG channel of the heterostructure was based on a strained In_1-X_Ga_X_As layer, whose thickness and composition were optimized by calculation and experimentation to prevent the generation of misfit dislocations, which is necessary to achieve high electron mobilities and sheet concentrations. Similarly, an appropriate doping level was found to fill the 2DEG channel with electrons and inhibit the formation of a parallel conducting channel with a low electron mobility in the delta doped layer. The strained In_1-X_Ga_X_As layer was grown close to the center of the heterostructure to avoid a significant bowing of the heterostructure nanomembrane during and after separation from the growth substrate.

The heterostructure was grown using low-pressure MOVPE in an Aixtron AIX 200 reactor (Aixtron, SE Dornkaulstr. 2, 52134 Herzogenrath, Germeny) at 700 °C. Hydrogen was used as the carrier gas. The precursors were trimethylgallium, trimethylindium, trimethylaluminum, arsine, and diluted silane.

The heterostructure consisted of a GaAs (001) substrate, 300 nm AlAs sacrificial layer, GaAs buffer layer, 10 nm 2DEG In_0.23_Ga_0.77_As channel, 4 nm Al_0.3_Ga_0.7_As spacer, delta-doped Si layer, 30 nm Al_0.3_Ga_0.7_As top layer, and 5 nm GaAs cap layer. The delta-doped Si layer was prepared during a 30 s growth interruption under an overpressure of arsine. The growth rates of AlAs, GaAs, In_0.23_Ga_0.77_As, and Al_0.3_Ga_0.7_As were 0.29, 0.17, 0.2, and 0.23 nm/s, respectively. The heterostructure was designed to have a nominal sheet electron concentration of 2.10^12^ cm^−2^. The as-grown heterostructure exhibited a sheet electron concentration of 3.4 × 10^12^ cm^−2^ and Hall mobility of 4590 cm^2^V^−1^s^−1^.

## 3. Results and Discussion

### 3.1. Separation

**Figure 1:**
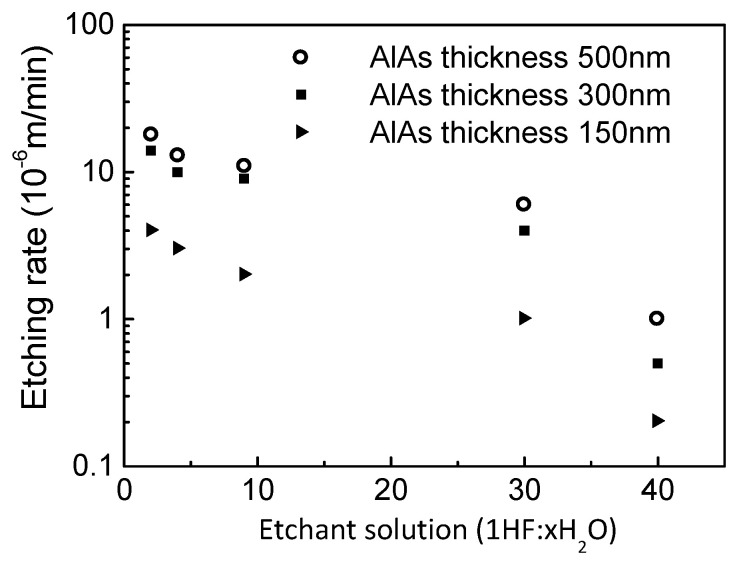
Dependence of the release etching rate of HEMT heterostructure nanomembranes on the composition of an HF-based solution with the thickness of the AlAs sacrificial layer as the parameter.

A III-V device structure can be separated from its growth substrate by a technique called epitaxial lift-off. It involves the selective lateral wet etching of a thin sacrificial layer. Once the layer is completely etched away, the III-V structure becomes separated from the underlying layers and growth substrate [21,22]. We tailored this technique to detach our HEMT structures from GaAs substrate. The rate of the heterostructure release was studied with dependence on the composition of an etchant based on hydrofluoric acid (HF). The thickness of the AlAs interlayer was the parameter (three values), and the etching was carried out at room temperature. The results are presented in Figure 1.

**Figure 2:**
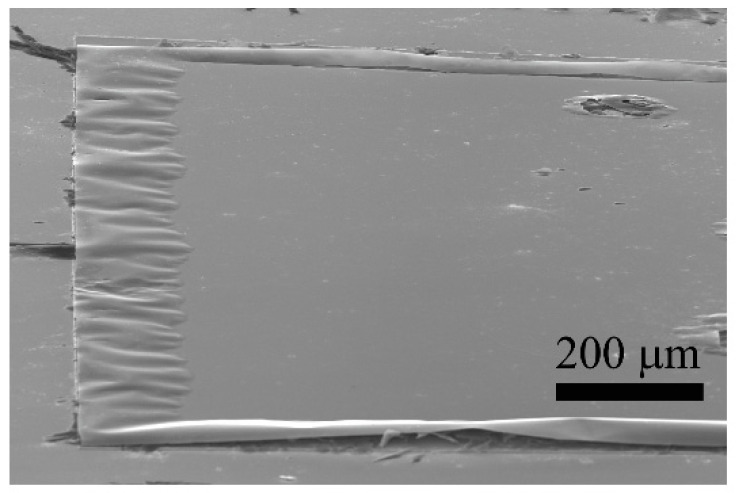
SEM image of a sample where the sacrificial etching of a 20 nm thick AlAs separation layer was self-terminated.

Thin AlAs layers (<100 nm) were not conducive to the HEMT heterostructure release because the sacrificial etching self-terminated at short distances from the edges of lithographically defined nanomembrane areas (a similar outcome was published previously) [23]. The self-termination of the etching was attributed to a very low wettability of the channel formed as the result of the AlAs etching, as the separation between the underlying GaAs buffer layer and the HEMT heterostructure was too narrow. The situation where the etching of the sacrificial layer (thickness of AlAs is 20 nm) was stopped at a short distance is documented in Figure 2. The wettability problem was alleviated by adding a small amount of a hydrophilic agent to the etching solution.

Nanomembranes of the HEMT heterostructure that were processed and studied in this experiment were 1.5 × 1.5 cm^2^ in size. Their release was achieved by the sacrificial etching of a 300 nm thick AlAs layer. To assure a successful transfer of the nanomembranes, it was necessary to have all surfaces of the nanomembranes as smooth and clean as possible. This would guarantee that the heterostructure adhered well to the host substrate by means of van der Waals forces, especially the surface that was exposed by the sacrificial etching at the original interface between the GsAs layer; the heterostructure had to be clean from any remnants of the AlAs sacrificial etching. To achieve this, the AlAs layer had to have very sharp interfaces with the overlying HEMT heterostructure and underlying GaAs buffer layer.

**Figure 3:**
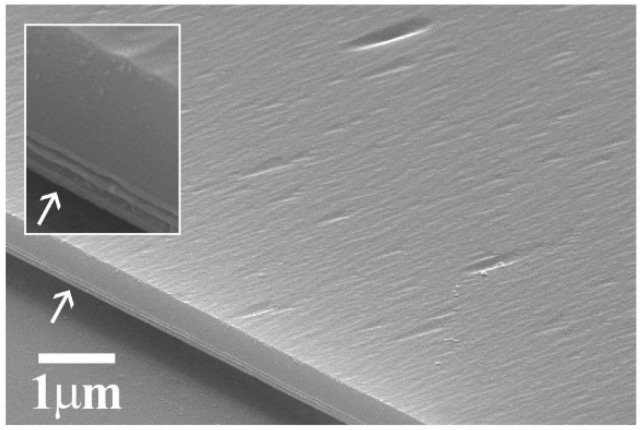
SEM images of an HEMT nanomembrane that was flipped over to expose the surface of its GaAs back side. It contained a shallow wavy structure with prolonged shallow depressions. The arrows mark an edge of the nanomembrane with the HEMT heterostructure delineated.

To look at the bottom surface of the nanomembranes, some were flipped over and attached upside down to sapphire host substrate, as is shown in the SEM micrographs of Figure 3. The white arrows in the main picture and the inset point to the edge of an HEMT nanomembrane where the heterostructure was delineated. The main SEM image shows the back side of a nanomembrane surface, which is that of the GaAs layer, after the sacrificial AlAs layer was completely etched away. This surface was originally the interface between the GaAs layer and the AlAl sacrificial layer, and it should ideally be completely flat and smooth. However, the SEM image revealed that it was not fully smooth but contained a shallow wavy structure with prolonged shallow depressions. The relatively large shallow depressions may have originated due to etching at crystal defects. The shallow wavy structure may have stemmed from the intermixing of Al and Ga atoms as the epitaxial growth was switched from AlAs to GaAs. As a result, a very narrow inhomogeneous AlGaAs layer was formed. As the HF-based etchant etched the AlAs layer away during the release process, it reached this inhomogeneous intermixed layer, etched it off (although more slowly compared with the AlAs layer), and left the shallow surface imprint behind. We believe that it did not significantly hamper the bonding of the nanomembranes to the sapphire substrate.

**Figure 4:**
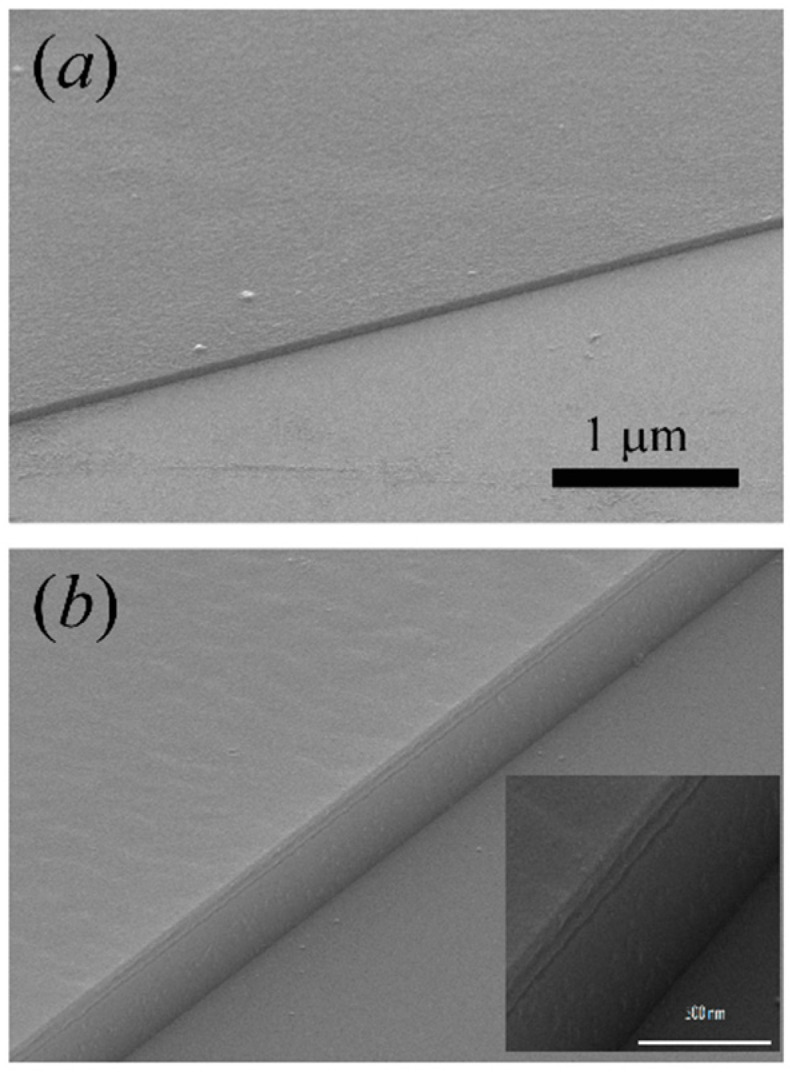
(**a**) SEM image of a GaAs nanomembrane attached to the host sapphire substrate by van der Waals forces. (**b**) SEM close-up of the transferred heterostructure nanomembrane with sharp interfaces between the layers.

Figure 4a shows an SEM image of a GaAs nanomembrane attached to the host sapphire substrate. The nanomembrane evidently adhered well to the substrate. Figure 4b shows a detail of the transferred heterostructure nanomembrane with sharp interfaces between the layers of the heterostructure.

Once the nanomembranes became separated from the growth substrate, the etching solution was carefully diluted with water. The membranes were then taken out of the solution and transferred to sapphire substrate. They were dried and thoroughly cleaned in preparation for HEMT processing. Van der Waals forces between the HEMT nanomembranes and sapphire were strong enough for the subsequent processing steps.

### 3.2. XRD Analysis

Successful production of final devices required the growth of high-quality heterostructure layers with smooth interfaces. To verify the structural quality of our layers, HRXRD measurements were performed on the HEMT heterostructure (including the AlAs sacrificial layer) grown on GaAs substrate. To assess the effect of release and transfer procedures on the quality of the heterostructure, the HRXRD measurement was repeated after sticking the structure to a host sapphire substrate. The X-ray analysis was performed in high-resolution mode with a Bruker D8 DISCOVER diffractometer(Bruker AXS Advanced X-ray Solutions GmbH, Östliche Rheinbrückenstraße 49, 76187 Karlsruhe, Germany) equipped with a rotating Cu anode that was operated at 12 kW (40 kV/300 mA). A parabolic Goebel mirror and a Bartels monochromator were inserted into the primary beam. Standard angular 2θ/ω scans were recorded to determine the composition and thickness of the layers. Linear scans in reciprocal space were performed to evaluate the degree of relaxation of the layers.

**Figure 5:**
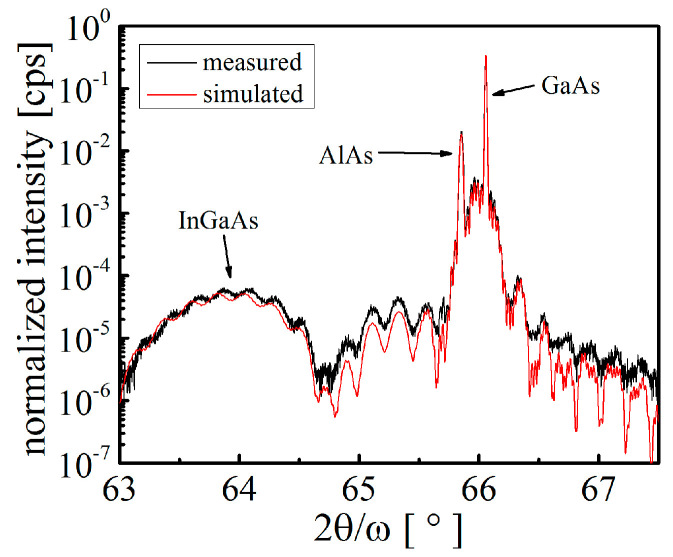
Comparison of the measured and simulated 2θ/ω curves.

The structures were analyzed by measuring high-resolution 2θ/ω curves of the 004 diffraction. The thickness and composition of the layers were determined by simulation of the theoretical curves using LEPTOS 3.04 software (provided by Bruker Company). Results of the X-ray measurements are exemplified in Figure 5, which compares the measured and simulated 2θ/ω curves. The measurements show that the diffraction maxima corresponding to the InGaAs and AlAs layers are clearly distinguished, along with the GaAs substrate maximum. The thickness fringes are also well resolved, indicating a high quality of the analyzed heterostructure. To find out whether the layers of the heterostructure were relaxed, linear scans (not shown here) across the asymmetric 224 diffraction were performed in the perpendicular (*l* scans) and parallel (*h* scans) directions with respect to the sample surface, respectively. It was found that the *l* scan of the 224 diffraction perfectly coincided with the *l* scan of the corresponding symmetric 004 diffraction. The *h* scans across the 224 diffraction maxima were measured at the values of *l* coordinates corresponding to the InGaAs and AlAs layers. The maxima of both curves were found to be precisely at h=2.000. These results clearly indicated that the layers did not undergo any relaxation, and the in-plane lattice parameters of the layers were therefore equal to the bulk value of the GaAs lattice parameter. Both InGaAs and AlAs layers were laterally strained, and the estimated values of the strain were ~−1.5% and ~−0.15%, respectively. These values were used as the input parameters in the simulation process of the theoretical 2θ/ω curves.

**Figure 6:**
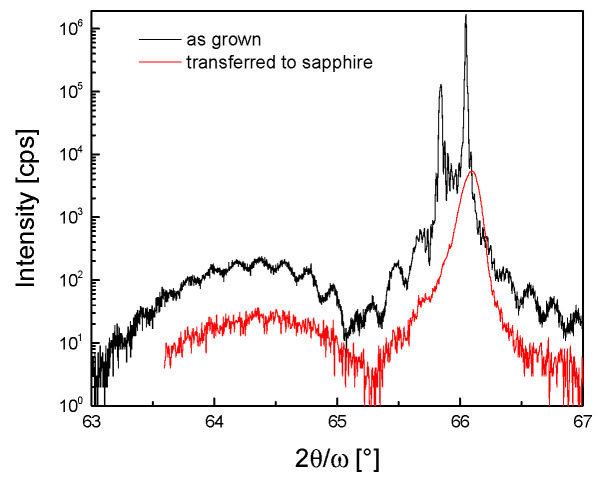
X-ray diffraction of the as-grown AlGaAs/InGaAs/GaAs heterostructure (with the AlAs layer) and of the same heterostructure after being released and fixed to sapphire.

The 2θ/ω curve of the 004 diffraction of the heterostructure fixed to the host sapphire substrate is shown in Figure 6. Only the maxima of the GaAs buffer layer and of the ~10 nm thick InGaAs layer were visible, the AlAs separation layer was removed at the releasing step, and the corresponding maximum was missing. The most pronounced feature of the diffraction curve is the overall decrease in intensity by about two orders of magnitude with respect to the initial heterostructure on GaAs substrate (Figure 6). This can be partially ascribed to the size of the sticked heterostructure. The dimension of the measured sample in this experiment was only ~1 mm^2^. The second effect influencing intensity is the possible loss of the planarity of the heterostructure that cannot be avoided during fine manipulation of the sample. The broadening of the GaAs diffraction maximum and disappearance of the thickness fringes seem to support this reasoning. Generally, X-ray diffraction is extremely sensitive to any distortion of the diffracting area. Hence, a slight bowing of the sample, which has no effect on the functionality of an electronic device, can seriously disturb the interference of X-rays and can result in the observed changes in the diffraction curve.

The X-ray measurements revealed that the heterostructures prepared on GaAs substrates were of very good quality with smooth interfaces. The release and transfer procedures did not damage the crystalline structure of the heterostructures and the main features of their diffraction curves were preserved.

### 3.3. PL Analysis

**Figure 7:**
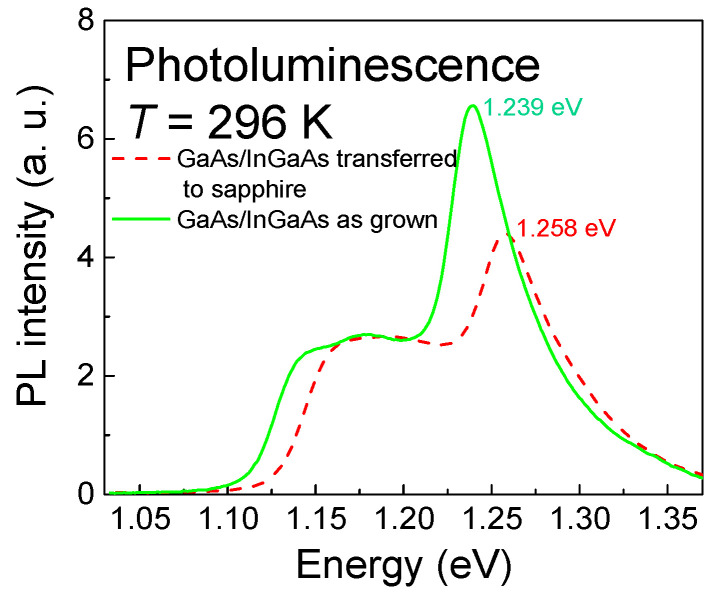
PL spectra of the as-grown heterostructure and released heterostructure nanomembrane.

The quality of the release and transfer of the heterostructure were also investigated using PL measurement at room temperature. A 488 nm line of argon ion laser served for the sample pumping. PL radiation from the sample was filtered via a quarter-meter monochromator (Monochromator DIGIKROM 240, CVI Laser Corporation, Albuquerque, NM, USA.) and detected by a liquid-nitrogen-cooled InGaAs photodiode. The detector signal was amplified and recorded by a standard lock-in technique. At first, we measured the PL signal of the as-grown heterostructure on the parent substrate. The measurement was subsequently repeated when the heterostructure was released and transferred to sapphire. Figure 7 compares both PL signals.

The figure shows that the PL peaks of the as-grown heterostructure shifted after the heterostructure nanomembrane was released and fixed to the sapphire substrate. Each PL spectrum exhibits two resonances that consist of an asymmetric broadened feature at the lower-energy side and a distinct feature at the higher-energy side. According to [24], the broadened feature at lower energies, designated as 11H, originates in optical recombination from the first conduction subband to the first heavy-hole valence subband. The distinctive feature at higher energies, labeled as 21H, results from the recombination of electrons in the second electron subband and optically excited holes. We observed a shift of 19 meV between the measured peaks. The energy shift can be explained with a re-distribution of charge in the nanomembrane. A part of the charge occupies deep levels at the bottom surface of the nanomembrane. This surface was originally the interface between the bottom GaAs layer and the sacrificial AlAs layer; it was exposed as the nanomembrane was released from the growth substrate. This leads to a change in the electric field intensity in the quantum well of the nanomembrane; hence, the PL transition energy is also changed. As the quantum well (QW) lost some of its charge, the conduction and valence band energies of InGaAs were decreased near the bottom InGaAs/GaAs interface. A lower electron concentration in the QW of the nanomembrane corresponds with the electrical characteristics of the transistors described below.

### 3.4. HEMT Processing

The HEMTs were processed by standard photolithographic techniques using AZ 5214E positive tone photoresist. At first, MESA etching was performed in an Oxford PlasmaLab apparatus (OxfordInstruments GmbH, Borsigstrasse15a, Wiesbaden, D 65205 Germany) using SiCl4. The etching was stopped on the sapphire substrate. Ohmic contact metallic layers based on Ni/90 nm AuGe /Ni were deposited through a photoresist mask. The NiGe layer was evaporated to achieve a eutectic alloy of 88% Ni and 12% Ge. The layers were lifted off and annealed at 450 °C in N2 for 30 s. The ohmic contacts exhibited a contact resistance of 0.3 Ωmm. The gate electrode (prepared using similar lithographic steps) was composed of non-alloyed 15nmTi/30nmPt/50nmAu layers.

**Figure 8:**
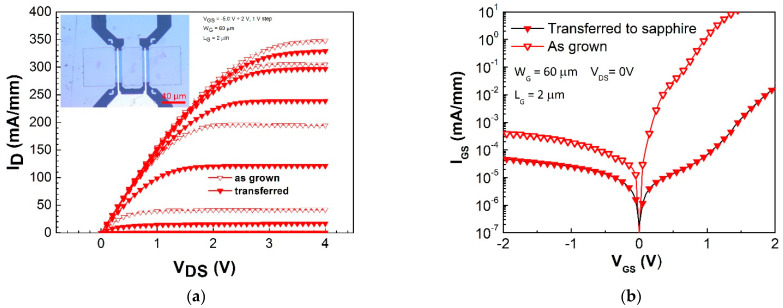
(**a**) Output characteristics of transistors processed on an HEMT heterostructure nanomembrane transferred to sapphire compared with those of transistors prepared on the as-grown heterostructure on GaAs substrate. The inset shows an image of an HEMT on the host substrate. (**b**) Comparison of the gate leakage current in two terminal measurement characteristics of HEMTs on sapphire substrate with those of HEMTs on GaAs substrate.

HEMTs were simultaneously processed on the growth GaAs substrate and host sapphire substrate for comparison. Basic dc transistor properties were measured. The output characteristics of both types of HEMT suggest that no degradation in the HEMT properties occurred. The input two-terminal measurement showed that the HEMTs processed on the heterostructure nanomembrane transferred to sapphire exhibited slightly decreased leakage current. The results are in Figure 8.

## 4. Conclusions

A 100 nm 2DEG AlGaAs/InGaAs/GaAs HEMT heterostructure was grown by MOCVD on GaAs substrate, released by HF: H_2_O epitaxial lift-off through a 300 nm AlAs layer, transferred, and attached to sapphire by van der Waals forces. 

The heterostructure contained a strained 10 nm 2DEG In_0.23_Ga_0.77_As channel with a sheet electron concentration of 3.4 × 10^12^ cm^−2^ and Hall mobility of 4590 cm^2^V^−1^s^−1^. This channel layer was grown close to the center of the heterostructure to suppress a significant bowing of the heterostructure nanomembrane during and after separation from the growth substrate.

The nanomembrane release rate was studied with respect to the thickness of AlAs (varied between 20 to 500 nm) and to the composition of HF: xH_2_O (for x varied between 2 to 40) at RT. Thin AlAs layers (<100 nm) did not facilitate the release of the nanomembrane as the etching of AlAs self-terminated.

The as-grown heterostructure and nanomembranes attached to sapphire were characterized by HRXRD, PL, and SEM. The InGaAs and AlAs layers were laterally strained: ~−1.5% and ~−0.15%. HRXRD showed that the as-grown heterostructure had very good quality and smooth interfaces, and the transferred nanomembrane had its crystalline structure and quality preserved. PL showed that the nanomembrane peak was shifted by 19 meV towards higher energies with respect to that of the as-grown heterostructure. 

The 2DEG channel transport properties were measured using HEMTs processed on the as-grown heterostructure and on 1.5 × 1.5 cm^2^ nanomembranes attached to sapphire. The nanomembrane HEMTs showed no degradation of the output characteristics and their input two-terminal measurement confirmed a slight decrease in leakage current.

This work demonstrated that the properties of the HEMTs were not adversely affected by the transfer of the HEMT heterostructure to sapphire. This is very promising for our follow-up work aimed at the hybrid integration of III-V- and III-N-based devices, in which transistors with excellent dc and high-frequency properties will be attached to host substrates [25].

## Data Availability

Not applicable.
